# The Pediatric Sepsis Biomarker Risk Model (PERSEVERE) Biomarkers Predict Clinical Deterioration and Mortality in Immunocompromised Children Evaluated for Infection

**DOI:** 10.1038/s41598-018-36743-z

**Published:** 2019-01-23

**Authors:** L. Jacobs, Z. Berrens, E. K. Stenson, M. W. Zackoff, L. A. Danziger, P. Lahni, H. R. Wong

**Affiliations:** 1 Division of Pediatric Critical Care, Texas Children’s Hospital, 6651 Main St. E1470.36, Houston, TX 77030 USA; 2 Division of Pediatric Critical Care, Riley Hospital for Children at the University of Indiana Health, 705 Riley Hospital Dr., Indianapolis, IN 46202 USA; 3 Division of Pediatric Critical Care, Cincinnati Children’s Hospital Medical Center, 3333 Burnet Ave MLC 2005, Cincinnati, OH 45229 USA; 4 Division of Pediatric Infectious Disease, Cincinnati Children’s Hospital Medical Center, 3333 Burnet Ave MLC 7017, Cincinnati, OH 45229 USA

## Abstract

Pediatric sepsis and bacterial infection cause significant morbidity and mortality worldwide, with immunocompromised patients being at particularly high risk of rapid deterioration and death. This study evaluated if PERSEVERE, PERSEVERE-II, or the PERSEVERE biomarkers, can reliably estimate the risk of clinical deterioration and 28-day mortality among immunocompromised pediatric patients. This is a single-center prospective cohort study conducted from July 2016 through September 2017 incorporating 400 episodes of suspected bacterial infection from the inpatient units at Cincinnati Children’s Hospital Medical Center, a large, tertiary care children’s hospital. The primary analysis assessed clinical deterioration within 72 hours of evaluation for infection. Secondarily, we assessed 28-day mortality. Clinical deterioration was seen in 15% of subjects. Twenty-eight day mortality was 5%, but significantly higher among critically ill patients. Neither PERSEVERE nor PERSEVERE-II performed well to predict clinical deterioration or 28-day mortality, thus we derived new stratification models using the PERSEVERE biomarkers with both high sensitivity and negative predictive value. In conclusion, we evaluated previously validated biomarker risk models in a novel population of largely non-critically ill immunocompromised pediatric patients, and attempted to stratify patients based on a new outcome metric, clinical deterioration. The new highly predictive models indicate common physiologic pathways to clinical deterioration or death from bacterial infection.

## Introduction

Sepsis remains a significant health issue worldwide, contributing to substantial morbidity and mortality among pediatric patients. Recent literature suggests an 8% point prevalence of pediatric severe sepsis, with an associated 25% in-hospital mortality rate^1^. Immunocompromised pediatric patients are at even higher risk of infection and subsequent deterioration, with mortality approaching 40–50%^1^. A 2015 epidemiologic study evaluating the incidence of infection among bone marrow transplant recipients indicated that almost half (46%) of patients have a documented infection during a hospital stay, with mortality rates doubling once a patient becomes infected^2^.

This burden has pushed researchers to seek a better understanding of individual patients’ phenotypes and risk of decompensation. Acquisition of patient-specific knowledge allows clinicians to identify patients at high risk of deterioration, better focus resources, and provide targeted therapies. The Pediatric Sepsis Biomarker Risk Model (PERSEVERE)^3^ is a biomarker-based stratification tool to estimate a specific patient’s baseline risk of mortality from sepsis. Using genome-wide expression profiling, Wong *et al*. identified twelve candidate prognostic biomarkers in critically ill septic pediatric patients^4–7^. The twelve biomarkers were pared down to five having the highest predictive capacity: interleukin-8 (IL-8), C-C chemokine ligand 3 (CCL3), heat shock protein 70 kDa 1B (HSPA1B), granzyme B (GZMB), and matrix metalloproteinase 8 (MMP-8)^3^. When combined with age, they yielded PERSEVERE, a highly sensitive predictor of mortality with 99% negative predictive value^3^. PERSEVERE has been tested in several cohorts and reliably estimates mortality risk among children with septic shock^8–10^. PERSEVERE-II combines platelet count with the five biomarkers to yield a mortality risk model that might have broader applicability across a wide range of sepsis phenotypes^8^.

The PERSEVERE models were developed exclusively among patients admitted to the intensive care unit (ICU) and meeting criteria for septic shock. It is unknown whether these models can estimate the risk of deterioration among non-ICU patients suspected of being bacterially infected at the time of suspicion for infection. This is conceptually important because risk models often perform differently when applied to cohorts that are clinically different than the original cohorts in which the models were derived. Here, we sought to expand the application of these previously validated biomarker models to a novel patient population in a non-ICU setting, and to a different outcome metric. Our primary aim was to determine if PERSEVERE, PERSEVERE-II, or the PERSEVERE biomarkers, can reliably estimate the risk of clinical deterioration within 72 hours of the initial suspicion for bacterial infection among immunocompromised pediatric patients. Secondarily, we determined if the models or biomarkers reliably estimate the risk of 28-day mortality in this population.

## Study Design and Methods

### Study Design

The study protocol was approved by the Institutional Review Board of Cincinnati Children’s Hospital Medical Center (CCHMC). This was a prospective study following a cohort of patients admitted to Cincinnati Children’s Hospital Medical Center (both general medical and surgical wards and intensive care units) between July 2016 and September 2017 with clinical suspicion for infection, defined by the acquisition of a blood culture at any point during the admission by the primary team, performed independently without interference from the research team. Upon enrollment, GZMB, HSPA1B, IL-8, CCL3, and MMP-8 levels were measured from blood collected at the time of concern for infection.

### Patients and Data Collection

Patients were included if they were admitted to CCHMC, met at least one criterion within the definition of immunocompromised (Table 1), had bacterial cultures sent during admission, and had a residual laboratory sample (a specimen in the clinical laboratory that would otherwise be discarded) available that was drawn within six hours of the qualifying blood culture. Patients were excluded if they did not meet the definition of immunocompromised or did not have a residual laboratory sample. No age cutoffs were applied. A waiver of consent was granted by the institutional review board as there was no more than minimal risk to the subjects, and no direct subject contact. Subjects were recruited using the electronic medical record for the institution. If more than one residual sample was available, the one drawn temporally closest to the blood culture was used. Patients could be enrolled in the study more than once, depending on if more than one blood culture was sent during admission. Subsequent blood cultures and associated residual samples were collected at least 24-hours from prior samples utilized. Thus, each subject could account for more than one episode in the study, defined as any new concern from the primary team for infection, as evidenced by acquisition of a new blood culture.

**Table 1:** Definition of Immunocompromised.

Neutropenia (ANC < 0.5 K/mcL)
Exposure to Chemotherapeutic Agent within prior 7 days
Exposure to Myeloablative Radiation within prior 7 days
Receipt of Solid Organ Transplant and Exposure to Immunosuppression^a^ within prior 7 days
Receipt of Bone Marrow Transplant and Exposure to Immunosuppression^a^ within prior 7 days

^a^Immunosuppression includes: high dose steroids (≥2 mg/kg/day methylprednisolone or equivalent), calcineurin inhibitor, anti-proliferative agent (i.e. mycophenolate or azathioprine), mTOR inhibitor (i.e. sirolimus), monoclonal antibody, thymoglobulin.

Clinical and demographic data were collected using the electronic medical record. Demographic data included age, gender, co-morbidities, reason for immunocompromised status, physical location of the patient at the time of specimen collection, and whether location changed within 72 hours. Clinical data included results of microbiological data (blood, urine, cerebrospinal fluid, pleural, peritoneal, and respiratory cultures), complete blood counts, basic metabolic profiles, liver function tests, relevant radiologic testing, as well as information on respiratory support, need for vasoactive medications, fluid requirements, changes in neurological status, and survival at 28 days.

### Main Outcome Measures and Primary Analysis

Our primary outcome of interest was clinical deterioration within 72 hours of evaluation for possible bacterial infection. We also evaluated mortality within 28 days of being evaluated for possible infection as a secondary outcome measure. Clinical deterioration was defined as a significant change in clinical status, including transfer to an intensive care unit, severe respiratory insufficiency or respiratory failure (initiation of high flow nasal cannula, non-invasive positive pressure ventilation, or intubation), administration of ≥60 mL/kg (or ≥3 L if ≥50 kg) of crystalloid in a 24 hour period, initiation of vasopressors or inotropic agents, altered mental status, or death (Table 2). Subjects met the definition of clinical deterioration if they fulfilled any one of the above criteria.

**Table 2:** Definition of Clinical Deterioration within 72 Hours.

Fulfillment of Any of the following criteria:
Transfer to an ICU (if originally located in a non-ICU)
Initiation of High Flow Nasal Cannula, Non-Invasive Positive Pressure Ventilation or Invasive Positive Pressure Ventilation
Receipt of ≥60 mL/kg of crystalloid if <50 kg OR ≥3 L of crystalloid if ≥50 kg
Initiation of Inotropic of Vasopressor Support (epinephrine, norepinephrine, vasopressin, phenylephrine, milrinone, dopamine)
New Onset Altered Mental Status (significant decline in GCS, somnolence, seizure)
Death

The primary analysis focused on the ability of PERSEVERE and PERSEVERE-II to estimate the risk of the two outcomes of interest. In the event that PERSEVERE and PERSEVERE-II did not perform well, we planned, *a priori*, to derive new models using the PERSEVERE biomarkers, age, and platelet count to estimate the risk of the two outcomes of interest.

### Study Procedures

Residual samples obtained from the CCHMC clinical laboratory were used to measure serum protein concentrations of the five biomarkers using a multiplex magnetic bead platform (MILLIPLEX™ MAP) designed by the EMD Millipore Corporation (Billerica, MA), and a Luminex® 100/200 System (Luminex Corporation, Austin, TX).

### Statistical Analysis

Data were described using medians and interquartile ranges, with individual biomarker performance evaluated by calculating the area under the receiver operating characteristic curves (AUROC’s). Comparisons between survivors and non-survivors, as well as those with and without clinical deterioration, were assessed using the Mann-Whitney *U*-test or χ-square test as appropriate. Using the previously derived PERSEVERE^3^ and PERSEVERE-II^8^ models, we assigned each subject a mortality probability, and thereafter calculated the AUROC’s for estimating the risk of clinical deterioration and 28-day mortality. The above analyses were performed using SigmaPlot version 13.0 software (Systat Software, Inc. San Jose, CA, USA). New decision trees were derived for risk of clinical deterioration and 28-day mortality using a classification and regression tree (CART) analysis, and taking into account the PERSEVERE biomarkers, age, and platelet count. The models were built using Salford Predictive Modeler version 6.6 (Salford Systems, San Diego, CA, USA). Decision tree performance characteristics were evaluated using the VassarStats website and are presented with their respective 95% confidence intervals.

### Ethics statement

All experimental protocols were approved by the institutional review board at Cincinnati Children’s Hospital Medical Center. The methods were carried out in accordance with the guidelines and regulations of said review board. As there was no direct patient contact, and no potential harm to subjects, the IRB granted a waiver of consent for this study.

## Results

### Cohort Characteristics

There were 293 subjects included in the study, yielding 400 episodes of suspected bacterial infection. Eighty-three subjects were enrolled more than once in the study, with 90% of enrollment episodes occurring at least ten days from that prior. The majority of subjects were re-enrolled after several weeks or months. The median age was 7.8 years (IQR 3.1–13.8 years). Fifty-three percent (n = 213) of the population had a primary oncologic diagnosis, 24% (n = 95) had received a bone marrow transplant, and 21% (n = 85) had received a solid organ transplant. Forty-eight percent (n = 193) of the cohort was neutropenic, and 48% (n = 190) were leukopenic. The overall infection rate was 37% (n = 148), with 70% of those patients having some form of culture positivity. The infection rate was highest among patients with solid organ transplants at 48%. The majority of subjects were in a non-ICU location at the time of suspicion for bacterial infection (n = 339), with 48 subjects residing in the PICU and 13 in the cardiac ICU at the time of enrollment. Among the patients initially evaluated in the general hospital wards, 7% (n = 25) required transfer to an ICU within 72 hours of study enrollment. Eight percent (n = 30/400) of the cohort had shock, defined as requiring significant fluid resuscitation, inotropic, or vasopressor support. Twenty-eight day mortality for the cohort was 5%, but was significantly higher among patients initially evaluated in an ICU (23%) compared to patients initially evaluated in a general ward (2%) (χ² (2, n = 400) = 41.2, p-value < 0.001).

### Clinical Deterioration

The clinical characteristics of the cohort are shown in Table 3, according to whether study subjects met criteria for clinical deterioration. There were no differences in median age, gender, or platelet count. The most common manifestations of clinical deterioration were new vasoactive requirement (27%), respiratory insufficiency or failure (26%), or transfer to an ICU (25%), followed by new altered mental status (13%), significant fluid resuscitation (11%), and death (1%). ICU patients were significantly more likely to demonstrate clinical deterioration with 52% deteriorating within 72 hours of enrollment compared with only 8% of non-ICU patients (χ² (2, n = 400) = 75.8, p-value < 0.001). Patients with a primary oncologic diagnosis or whom had undergone bone marrow transplant were less likely to show clinical deterioration, whereas those with solid organ transplants were more likely to deteriorate. Additionally, patients confirmed to have bacterial infection were more likely to show clinical deterioration than non-infected subjects (25% vs. 9%), and 62% of patients who clinically worsened were bacterially infected (χ² (2, n = 400) = 17.2, p-value < 0.001). Patients with evidence of clinical deterioration were significantly more likely to die within 28 days (20% mortality rate versus 3%) (χ² (2, n = 400) = 27.5, p-value < 0.001).

**Table 3:** Clinical Characteristics of Subjects with Stable Clinical Course vs. Clinical Deterioration.

	Stable Clinical Course (n = 340)	Clinical Deterioration (n = 60)	p-value
Age (years), median (IQR)	7.9 (3.6–13.7)	7.0 (1.5–14.8)	0.51
Female, %	46%	43%	0.06
Bone Marrow Transplant or Oncologic Diagnosis, %	74%	60%	0.03
Solid Organ Transplant, %	19%	35%	0.007
Non-ICU Location, %	91%	47%	<0.001
Platelet Count (K/mcL), median (IQR)	76 (33–184)	96 (38–214)	0.45
Bacterially Infected, %	33%	62%	<0.001
PERSEVERE Mortality Risk, median (IQR)	0.01 (0.01–0.01)	0.01 (0.01–0.18)	<0.001
PERSEVERE-II Mortality Risk, median (IQR)	0.007 (0.007–0.13)	0.09 (0.007–0.33)	<0.001
28-Day Mortality, %	3%	20%	<0.001

We assigned a PERSEVERE- and PERSEVERE-II-based mortality risk to each study subject, and assessed their ability to identify study subjects with subsequent clinical deterioration. Neither performed well, with the AUROC of PERSEVERE at 0.57 (0.51–0.63, p-value 0.05), and PERSEVERE-II at 0.63 (0.57–0.69, p-value 0.0003).

**Figure 1:**
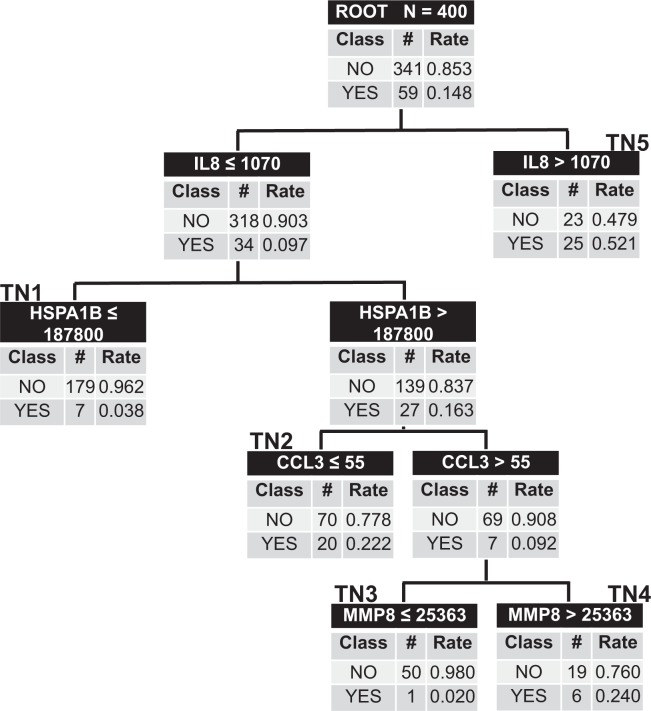
New Classification and Regression Tree to Predict Clinical Deterioration. The classification tree consists of four biomarker-based decision rules with five terminal daughter nodes. The tree incorporates four of five PERSEVERE biomarkers: interleukin-8 (IL8), heat shock protein 70 kDa 1B (HSPA1B), chemokine ligand 3 (CCL3), and matrix metalloproteinase-8 (MMP8). Each node denotes the number of subjects in the node, the serum concentration of a given biomarker determining the branch point (pg/mL), and both the total number and accompanying rate of subjects with clinical deterioration or clinical stability. Terminal Nodes 1 and 3 are considered low risk terminal nodes; terminal nodes 2, 4, and 5 are high risk. The AUROC for this model was 0.81.

We then attempted to derive a new model via classification and regression tree analysis, using the five PERSEVERE biomarkers, age, and platelet count as candidate predictor variables (Fig. 1). The root node included all 400 subjects with each subsequent daughter node arising from binary partitions of specific biomarkers. IL-8 concentration informed the first decision point with levels >1070 pg/mL yielding a high risk terminal node (terminal node 5) with 52% of subjects demonstrating clinical deterioration. The other higher risk nodes were terminal nodes 2 and 4 with 22% and 24% of patients demonstrating clinical deterioration, respectively. Subjects allocated to terminal nodes 3 and 1 were low risk with a 2.0–3.8% rate of clinical deterioration. The AUROC of this new model to predict clinical deterioration was 0.81 (0.76–0.87, p-value < 0.0001), with a sensitivity of 86% and negative predictive value of 97%. Further test characteristics can be found in Table 4. The ten-fold cross-validation AUC for the model was 0.72.

**Table 4:** Test Characteristics of the New Clinical Deterioration and New 28-Day Mortality Models.

	Sensitivity	Specificity	PPV	NPV	Positive LR	Negative LR
Clinical Deterioration Model	86% (74–94)	67% (62–72)	31% (24–39)	97% (93–98)	2.6 (2.2–3.2)	0.2 (0.1–0.4)
28-Day Mortality Model	100% (81–100)	69% (64–74)	15% (10–23)	100% (98–100)	3.2 (2.8–3.8)	—

*PPV* positive predictive value, *NPV* negative predictive value, *LR* likelihood ratio.

### 28-Day Mortality

The clinical characteristics of survivors and non-survivors at 28-days are displayed in Table 5. There were no differences between cohorts in median age, gender, primary diagnosis, or platelet count. A significantly higher proportion of ICU patients were deceased at 28-days: 23% versus 2% (χ² (2, n = 400) = 41.2, p-value < 0.001). As expected, more than 50% of non-survivors showed clinical deterioration within 72-hours of infectious evaluation, compared with just 13% of survivors (χ² (2, n = 400) = 27.5, p-value < 0.001).

**Table 5:** Clinical Characteristics of Survivors vs Non-Survivors.

	Survivors to 28-Days (n = 379)	Non-Survivors to 28-Days (n = 21)	p-value
Age (years), median (IQR)	7.9 (3.5–13.8)	5.4 (1.6–13.0)	0.21
Female, %	46%	43%	0.96
Bone Marrow Transplant or Oncologic Diagnosis, %	72%	71%	0.87
Solid Organ Transplant, %	27%	19%	0.98
Non-ICU Location, %	88%	33%	<0.001
Platelet Count (K/mcL), median (IQR)	81 (33–190)	57 (46–151)	0.82
Bacterially Infected, %	36%	57%	0.08
PERSEVERE Mortality Risk, median (IQR)	0.01 (0.01–0.01)	0 (0.01–0.01)	0.005
PERSEVERE-II Mortality Risk, median (IQR)	0.007 (0.007–0.17)	0.19 (0.007–0.33)	0.005
Clinical Deterioration, %	13%	57%	<0.001

**Figure 2:**
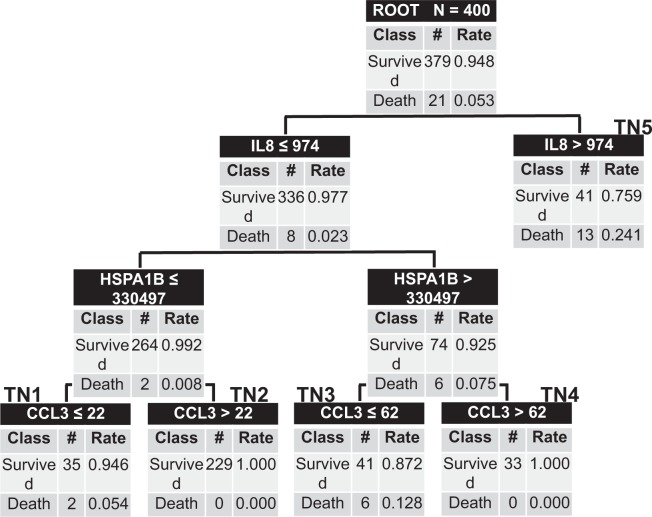
New Classification and Regression Tree to Predict 28-Day Mortality. The classification tree consists of four biomarker-based decision rules with five terminal daughter nodes. The tree incorporates three of five PERSEVERE biomarkers: interleukin-8 (IL8), heat shock protein 70 kDa 1B (HSPA1B), and chemokine ligand 3 (CCL3). Each node denotes the number of subjects in the node, the serum concentration of a given biomarker determining the branch point (pg/mL), and both the total number and accompanying rate of survivors and non-survivors. Terminal nodes 1, 2, and 4 are low risk nodes. Terminal node 3 is higher risk, with terminal node 5 being the highest risk node. The AUROC for this model was 0.87.

We further evaluated the utility of the PERSEVERE and PERSEVERE-II models to predict mortality at 28-days in this cohort of immunocompromised patients. As with clinical deterioration, the AUROC’s were low at 0.62 (0.46–0.79, p-value 0.06), and 0.67 (0.55–0.79, p-value 0.008), respectively. We again derived a new model in the same manner as previously described using the five PERSEVERE biomarkers, age, and platelet counts as candidate predictor variables (Fig. 2). The root node included all 400 subjects, and similar to the clinical deterioration model, IL-8 served as the first decision point. Terminal node 5 was the highest risk node, as 24% of subjects with IL-8 levels >974 pg/mL died by 28-days. Terminal nodes 1, 2, and 4 were low risk nodes with mortality ranging from 0–5%, and terminal node 3 was higher risk with 13% mortality. The AUROC of the new model to predict 28-day mortality was 0.87 (0.80–0.94, p-value < 0.0001). The summary ten-fold cross-validation AUC for the model was 0.77. The sensitivity of the model was 100%, and the negative predictive value was 100%. Further test characteristics can be found in Table 4.

## Discussion

PERSEVERE and PERSEVERE-II are validated stratification tools to estimate baseline mortality risk among children with septic shock. These models have been derived and tested exclusively among critically ill patients with septic shock. We prospectively tested these tools in a cohort of largely general ward-based, immunocompromised pediatric patients. As the AUROC’s of both stratification tools were too low to have clinical utility, we used the five PERSEVERE biomarkers to derive new highly sensitive and predictive models, yielding novel applications for the PERSEVERE biomarkers.

We present the first use of the PERSEVERE biomarkers to prognosticate clinical deterioration. Undoubtedly, understanding a patient’s mortality risk is important, but perhaps more salient to a clinician is the ability to predict trajectory within the next several days. Knowing which patients are imminently likely to worsen encourages vigilance and earlier intervention. It is unsurprising that the AUROC’s to predict clinical deterioration for both PERSEVERE and PERSEVERE-II were lower in our population. The present cohort is vastly different than those in prior work: these mostly non-critically ill subjects started at a different place in their infectious course, 80% of deteriorating patients still remained alive at 28 days, and all were immunocompromised. The original models speak to mortality risk, and although the outcomes are biologically related, a model to predict clinical deterioration targets a different endpoint than one predicting mortality.

This is also the first testing of PERSEVERE to predict mortality risk in a non-ICU setting, among patients with bacterial infection but predominantly not in shock. Of the 400 subjects, only 15% resided in an ICU at the time of suspicion for infection, and less than 10% were in a state of shock. We assigned each patient a PERSEVERE- and PERSEVERE-II- derived mortality risk and although significantly different between survivors and non-survivors, the AUROC’s for clinically-relevant prognostication were low. This could be related to several factors. Age (PERSEVERE) and platelet count (PERSEVERE-II) were decision points in the original models but not in the new mortality model. PERSEVERE-II was recalibrated incorporating platelet count, and performed particularly well in a subset of septic patients with thrombocytopenia-associated multiple organ failure (TAMOF)^11–13^. Our analysis showed no differences in age or platelet count between survivors and non-survivors. Age likely played less of a role in our model since the range was so large (2 months to 38 years). In addition, almost 50% of our cohort was neutropenic and leukopenic, which could affect biomarker levels, albeit in unpredictable ways. In our prior work in this cohort, median procalcitonin (PCT) levels were significantly higher in lymphopenic patients and lower in neutropenic patients. In the present study, the biomarkers that created the first three decision points in the CART analysis (IL-8, HSPA1B and CCL-3) were both higher (IL-8 and CCL-3) and lower (HSPA1B) in lymphopenic and neutropenic patients. Finally, 28-day mortality rates were quite different between our cohort and those in earlier work. Five percent of the current population was deceased at 28-days, with mortality in prior studies ranging from 10–13%^3,8,9^, which could have impacted the test characteristics.

A major strength of the study is the design. This was a prospective cohort study drawing on a relatively large pediatric population, which generated granular data. This work focused on a particularly vulnerable group, immunocompromised patients, in which infection sets in more readily, and progresses with little resistance. This population would benefit from reliable prognostication, perhaps more than any other sub-group, as they often rapidly decline. Early knowledge of expected clinical trajectory and mortality risk could inform clinical decision making regarding triage, resource utilization, and therapy. By evaluating clinical deterioration, we also present a new avenue for application of the PERSEVERE biomarkers – a different outcome metric that can provide clinicians insight into the expected course over the next 72 hours.

There are two significant weaknesses of this study. The first is that we employed stringent criteria to define immunocompromised, which could have led to the exclusion of some high-risk subjects. The second weakness is the under-performance of the previously validated PERSEVERE and PERSEVERE-II models. But it is important to remember that cohort characteristics undoubtedly impact biomarker performance. Testing these stratification tools in subjects who are less sick, have lower mortality rates, and are starting from different points in the course of infection is likely to yield disparate results. Our data suggests that the PERSEVERE biomarkers reflect common pathways to deterioration or death from bacterial infection, but the way in which they inform us depends on a given patient’s starting point.

Despite the low AUROC’s associated with the original PERSEVERE-based models, using the same biomarkers in different classification and regression-tree based models to predict clinical deterioration and mortality yielded promising results. The new tools have very low false negative rates, an important feature for stratifying tests. Future work is required to prospectively validate the two new models.

## Conclusion

We evaluated the previously validated biomarker risk models, PERSEVERE and PERSEVERE-II, in several novel ways: to stratify patients based on a new outcome metric, clinical deterioration, among immunocompromised pediatric patients, and in a non-ICU setting. Although the original models had low AUROC’s to prognosticate about clinical deterioration and mortality, we were able to derive new highly sensitive and predictive models using the same variables in different regression patterns. This work represents an important step toward identifying at-risk infected pediatric patients whom require a heightened state of vigilance.

## Data Availability

The datasets generated during and/or analyzed during the current study are available from the corresponding author on reasonable request.
